# A prognostic model for acute myeloid leukemia based on ferroptosis-related lncRNA and immune infiltration analysis

**DOI:** 10.52601/bpr.2024.240029

**Published:** 2024-12-31

**Authors:** Shuhan Liu, Yingli Chen, Qianzhong Li, Zhiyu Fan, Menglan Li, Pengyu Du

**Affiliations:** 1 Laboratory of Theoretical Biophysics, School of Physical Science and Technology, Inner Mongolia University, Hohhot 010021, China; 2 The State Key Laboratory of Reproductive Regulation and Breeding of Grassland Livestock, Inner Mongolia University, Hohhot 010021, China

**Keywords:** Acute myeloid leukemia, Ferroptosis, Long non-coding RNA, Prognosis, Immune infiltrates

## Abstract

Acute myeloid leukemia (AML) is a rare tumor that invades the blood and bone marrow, it is rapidly progressive, highly aggressive, and difficult to cure. Studies have shown that long non-coding RNA (lncRNA) and ferroptosis play important roles in AML. However, few studies have been done on ferroptosis-related lncRNA for AML. To investigate the role of ferroptosis-related lncRNA in AML prognosis, we screened the differentially expressed genes related to ferroptosis and lncRNA. Ferroptosis-related lncRNA associated with AML prognosis was obtained by Pearson correlation analysis. By using univariate Cox analysis, least absolute shrinkage and selection operator (LASSO) analysis, and multivariate Cox analysis, the ten prognostic genes were used for constructing the prognostic model. The model was then validated using a Kaplan-Meier analysis and Cox regression analysis. The ROC results have shown that the model could better predict AML survival. We identified some mutated genes that may affect the poor prognosis based on the somatic mutation analysis. The enrichment pathway analysis of prognostic genes revealed that these genes were mainly enriched in some immune pathways and cancer pathways. By immune infiltration analysis, we found that high-risk patients may respond better to immunotherapy.

## INTRODUCTION

Acute myeloid leukemia (AML) is one of the most common types of leukemia in the family of leukemias. The disease is characterized by the impaired differentiation of myeloid progenitor/precursor cells and uncontrolled clonal expansion, leading to bone marrow failure and impaired hematopoiesis (Blum and Mims 2020). Despite the large advances in anti-leukemia drug mining (DiNardo and Wei 2020), it was difficult to significantly improve patient survival due to the complexity of the tumor microenvironment, the continuous evolution of dominant clones, and the intricacies of the molecular mechanisms (Marando and Huntly 2020). Therefore, searches for effective prognostic markers and potential targets for intervention are important for achieving personalized and precise diagnosis and treatment (Chen *et al.* 2023).

Ferroptosis is a novel type of cell death, distinct from autophagy and apoptosis (Wang *et al.* 2021). It depends on the changes of the levels of iron ions and reactive oxygen species in the cell, and leads to lipid peroxidation that regulates cell necrosis. Morphologically, ferroptosis is mainly manifested as a reduction in the mitochondrial size of cells, a decrease in mitochondrial cristae, and an increase in biofilm density, and in terms of cellular composition, ferroptosis is manifested as an increase in lipid peroxidation and the elevation of reactive oxygen species (ROS) (Marando and Huntly 2020). The effect of ferroptosis on cells is often a "double-edged sword", depending on the action of the cell and the environment in which the effect on the cell may be both to promote cell growth and to inhibit cell growth.

Long non-coding RNA (lncRNA) is a class of RNA molecules with more than 200 nucleotides characterized by the lack of an open reading frame (Ponting *et al.* 2009). They are important molecules in cell signaling *in vivo*, regulating gene expression and participating in various biological regulatory processes such as transcriptional silencing and chromosome modification (Wang and Chang 2011). With the in-depth study of lncRNA, it has been found that lncRNA plays key roles in the regulation of proliferation, metastasis, cell cycle, and programmed death in cancer (Jiang *et al.* 2021), among which the aberrant expression of ferroptosis-related lncRNA has been associated with cell proliferation, migration, tumorigenesis, and drug resistance in cancer, suggesting the importance of ferroptosis-related lncRNA in tumor therapy. However, the effect of ferroptosis-related lncRNA on AML still remains to be explored.

In this paper, we constructed a prognostic model for AML based on ten ferroptosis-related lncRNA and validated their role in AML prognosis. Since the prognosis of AML patients is closely related to mutation and immune microenvironment, we further explored the relationship between this feature and tumor mutation load and immune infiltration. It was validated that prognostic modeling may be useful for predicting the prognosis of AML patients and providing a theoretical basis for their subsequent treatment.

## RESULT

### Identification of differentially expressed ferroptosis-related lncRNA

**Figure 1:**
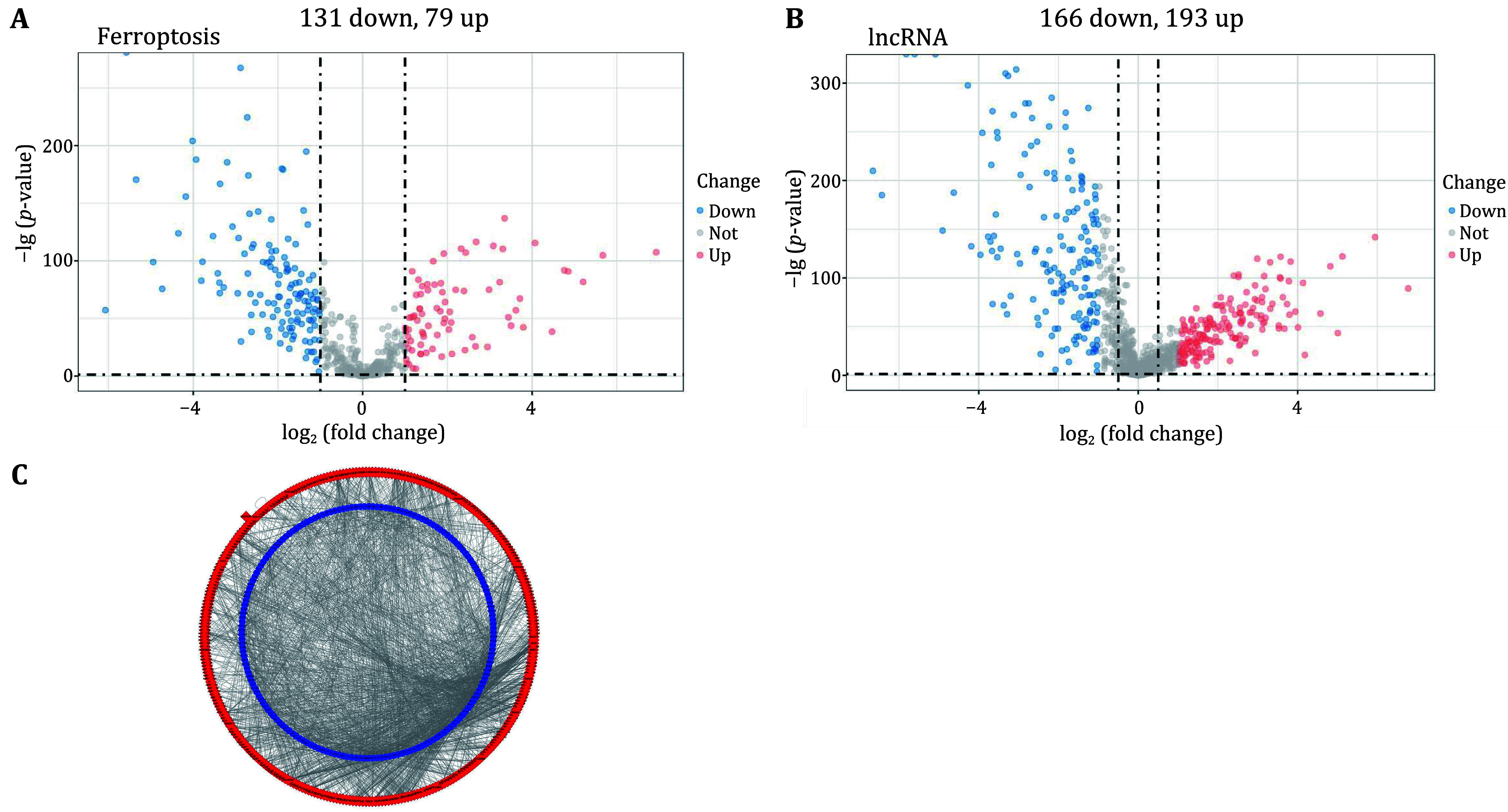
Identification of ferroptosis-related lncRNA. **A** Differential analysis of ferroptosis genes in AML. **B** Differential analysis of lncRNA in AML. **C** Correlation analysis between the differentially expressed ferroptosis genes and differentially expressed lncRNAs. Blue represents differentially expressed ferroptosis genes, red represents differentially expressed lncRNAs

The differential analysis yielded 131 down-regulated and 79 up-regulated genes, totaling 210 differentially expressed ferroptosis-related genes (Fig. 1A). The total number of differentially expressed lncRNA was 359, including 166 down-regulated and 193 up-regulated genes (Fig. 1B). Correlation analysis yielded a total of 317 ferroptosis-related lncRNAs (Fig. 1C).

### Construction of prognostic models in AML

**Figure 2:**
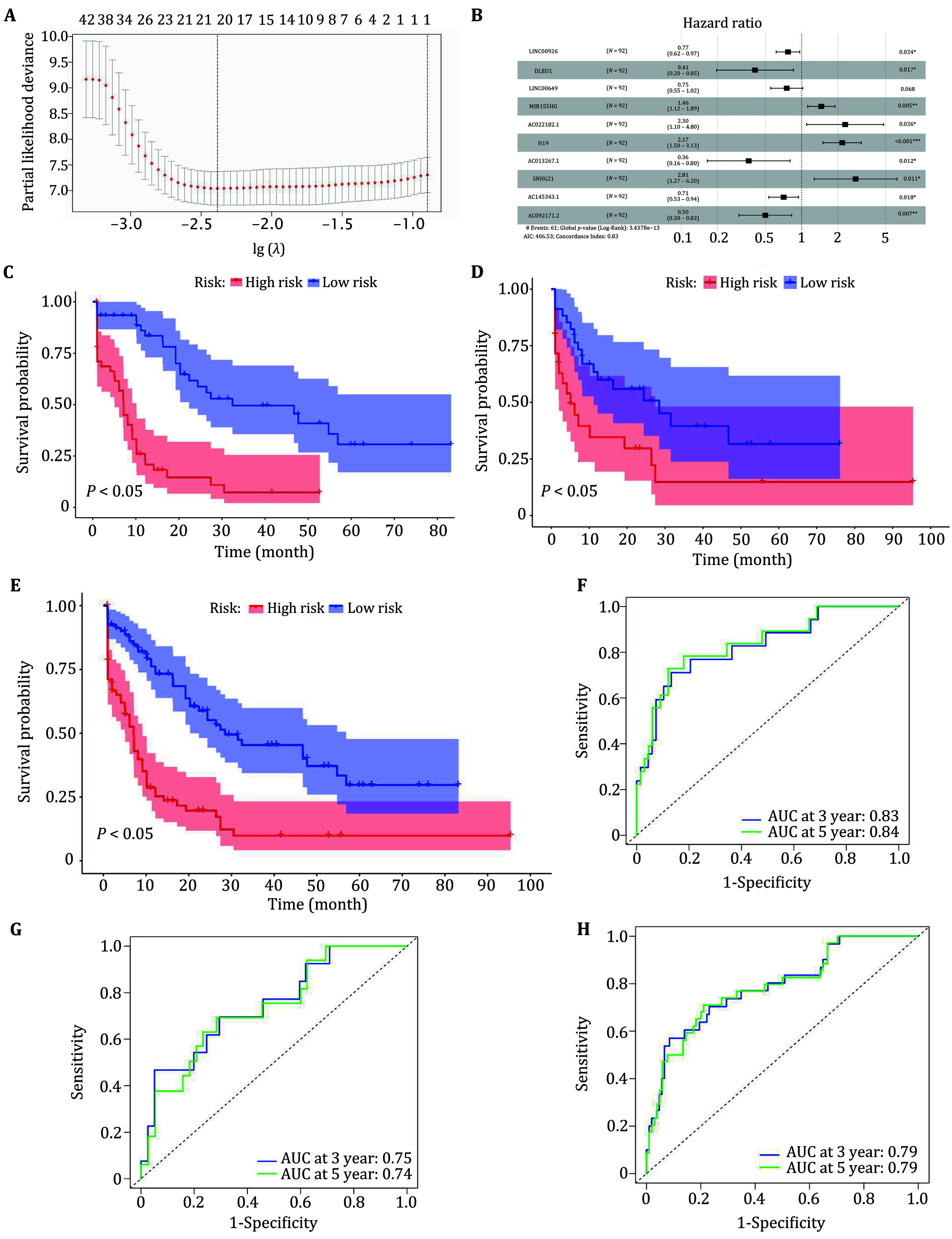


To examine the prognostic value of these ferroptosis-related lncRNA, we performed an initial screening based on the training set using univariate Cox analysis and obtained 102 prognostically relevant ferroptosis-related lncRNA (*p* < 0.05). Based on the results of the univariate Cox analysis, we screened out 20 ferroptosis-related lncRNA using LASSO regression analysis (Fig. 2A). Then we performed a multivariate Cox analysis to screen out ten ferroptosis-related lncRNA that were associated with prognosis in AML patients (Fig. 2B). Finally, we constructed a prognostic risk model based on the ten lncRNA, and the risk coefficients of each lncRNA in the model are shown in Table 1. The risk score formula was as follows:

**Figure 2:** Construction of ferroptosis-related lncRNAs prognostic modeling. **A** Identification of prognostic ferroptosis-related lncRNAs in AML patients with 10-fold cross-validation for variable selection in LASSO Cox regression. **B** Forest map with multivariate Cox regression analysis results. The horizontal line corresponds to the 95% confidence interval, and the vertical line indicates a C- index of 1. **C** KM survival curves for OS of patients in high and low risk groups in the Train set. **D** KM survival curves for OS of patients in high and low risk groups in the testing set. **E** KM survival curves for OS of patients in high and low risk groups in the whole set. **F** ROC curve of the model risk score in the training set. **G** ROC curve of the model risk score in the testing set. **H** ROC curve of the model risk score in the whole set

Y

**Table 1:** Risk coefficient values of ten lncRNA obtained by Multivariate Cox analysis

Gene	Coefficient
LINC00926	–0.260047824
DLEU1	–0.89052869
LINC00649	–0.284745146
MIR155HG	0.376009975
AC022182.1	0.832993922
H19	0.775326247
AC013267.1	–1.010812087
SNHG21	1.032770419
AC145343.1	–0.343010765
AC092171.2	–0.693727315

$ RS=\sum_{i \;=\; 1}^ncoef_i\times el_i, $

where *i* denotes the *i*-th lncRNA, *n* denotes the total number of related lncRNAs, *coef*_*i*_ denotes the regression coefficient and represents the contribution of lncRNA *i* to the prognostic risk score in the multivariate Cox analysis, and *el*_g_ denotes the expression level of the *i*-th gene (Li *et al.* 2023).

The risk scores were calculated by using the expressions and coefficients of the ten lncRNAs as follows:

\begin{equation*}\begin{split} 
RS=&-0.260047824\times {el}_{LINC00926}-0.89052869\times {el}_{\mathrm{D}\mathrm{L}\mathrm{E}\mathrm{U}1}\\&
- 0.284745146\times {el}_{\mathrm{L}\mathrm{I}\mathrm{N}\mathrm{C}00649}+0.376009975\\&
\times {el}_{\mathrm{M}\mathrm{I}\mathrm{R}155\mathrm{H}\mathrm{G}}+0.832993922\times {el}_{\mathrm{A}\mathrm{C}022182.1} \\&
+ 0.775326247\times {el}_{\mathrm{H}19}-1.010812087\times {el}_{\mathrm{A}\mathrm{C}013267.1}\\&
+1.032770419\times {el}_{\mathrm{S}\mathrm{N}\mathrm{H}\mathrm{G}21}-0.343010765\\&
\times {el}_{\mathrm{A}\mathrm{C}145343.1}-0.693727315\times {el}_{\mathrm{A}\mathrm{C}092171.2} .
\end{split}\end{equation*}

According to the median value of risk, the patients in the training set were divided into a high risk group and a low risk group, of which there were 46 patient samples in the high risk group and the low risk group, respectively. Kaplan-Meier survival curves were next drawn to compare the difference in overall survival (OS) between the high risk and low risk groups, which indicated that patients in the low risk group had better survival than those in the high risk group in the training set, testing set, and the whole set (Figs. 2C–2E). In addition, time-dependent ROC curves were plotted to assess 3-year and 5-year survival in AML patients. The AUC of 3-year and 5-year survival rates were predicted at 0.83, 0.84, and 0.75, 0.74, respectively, for the training set and the validation set. In the whole set, the predicted AUC value for 3-year and 5-year survival rates was 0.79 (Figs. 2F–2H). The above results indicate that the model is able to predict the prognosis of AML better.

### Independent prognostic analysis

**Figure 3:**
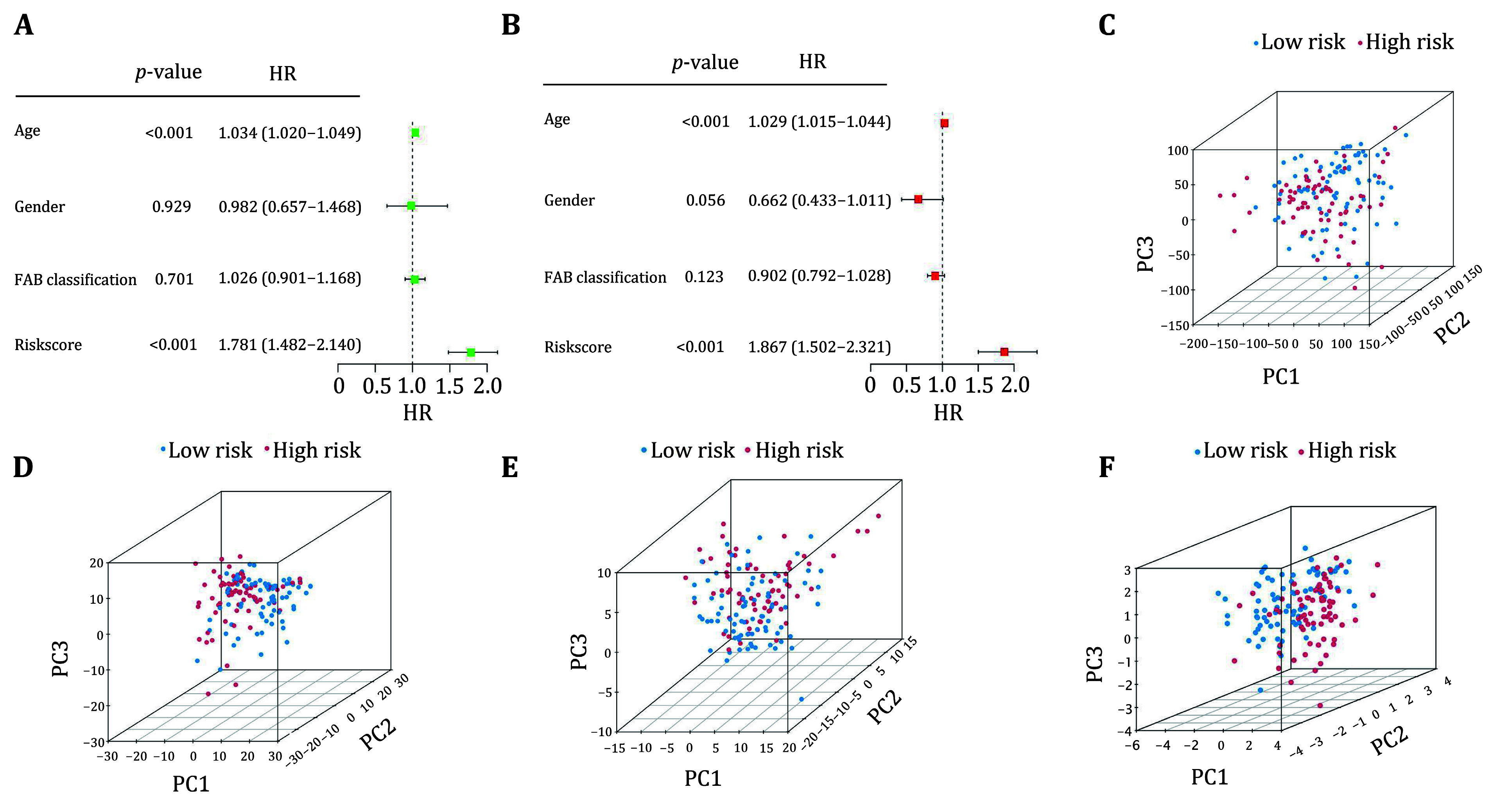
Independent prognostic role of the prognostic risk model for AML patients. **A** The correlations between the risk score and clinicopathological factors by univariate Cox regression analysis. **B** The correlations between the risk score and clinicopathological factors by multivariate Cox regression analysis. **C** PCA of all genes. **D** PCA of ferroptosis-related genes. **E** PCA of ferroptosis-related lncRNA genes. **F** PCA of prognostic genes

To validate the independent predictive ability of the prognostic model for AML patients, we performed univariate and multivariate Cox regression analyses, incorporating age, gender, FAB classification, and risk score as possible risk factors. In univariate Cox regression analysis, the HR for age was 1.034 with a 95% CI of 1.020–1.049 (*p* < 0.001) and the HR for risk score was 1.781 with a 95% CI of 1.482–2.140 (*p* < 0.001). In multivariate Cox regression analysis, the HR for age was 1.029 with a 95% CI of 1.015–1.044 (*p* < 0.001) and the HR for risk score was 1.867 with a 95% CI of 1.502–2.321 (*p* < 0.001). These results indicate that the prognostic model proposed in our study could serve as an independent prognostic factor for AML patients (Figs. 3A and 3B).

Then the grouping of the prognostic risk model was further analyzed by performing PCA for all genes, ferroptosis-related genes, ferroptosis-related lncRNA genes, and prognostic model genes. The results found that the sample distribution of all genes, ferroptosis-related genes, and ferroptosis-related lncRNA in the high and lowrisk groups were relatively dispersed, compared to the prognostic genes in the high and low risk groups, where the clustering of the samples for prognostic genes was better than the other PCA (Figs. 3C–3F). These results suggest that the prognostic risk model is an important independent prognostic risk factor for AML patients and can effectively distinguish between the low and high risk groups.

### Clinical subgroup analysis of risk models

**Figure 4:**
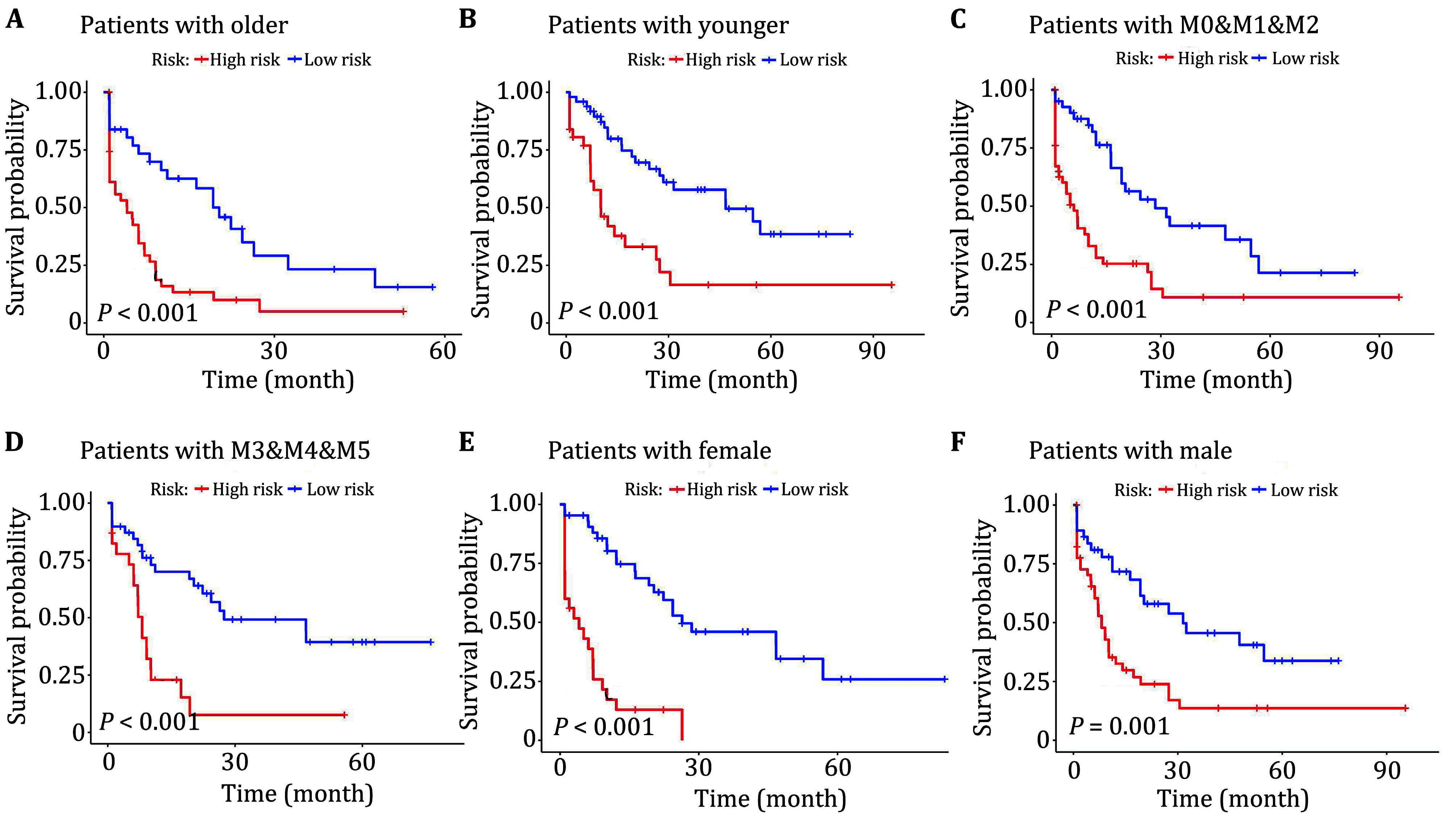
Clinical subgroup analysis. **A** older. **B** younger. **C** M0&M1&M2. **D** M3&M4&M5. **E** Female. **F** Male

In addition, the prognostic model's risk score was used as an indicator of clinical patients who were categorized into the high and low risk groups based on the median value. The clinical case factors were considered according to age, FAB typing, and gender. Age was defined as greater than 65 years for the older group and vice versa for the younger group. Due to the small number of individual samples, M0, M1, and M2 were grouped together, while M3, M4, and M5 were grouped together. Subsequently, KM survival analysis was conducted on patient OS based on age, FAB typing, and gender (Figs. 4A–4F). The results indicated that patients with high risk scores had shorter survival times compared to those with low risk scores, regardless of age, FAB typing, or gender. These results validate the reliability of the prognostic risk model and demonstrate its good predictive ability in different clinical subgroups.

### Mutation analysis

**Figure 5:**
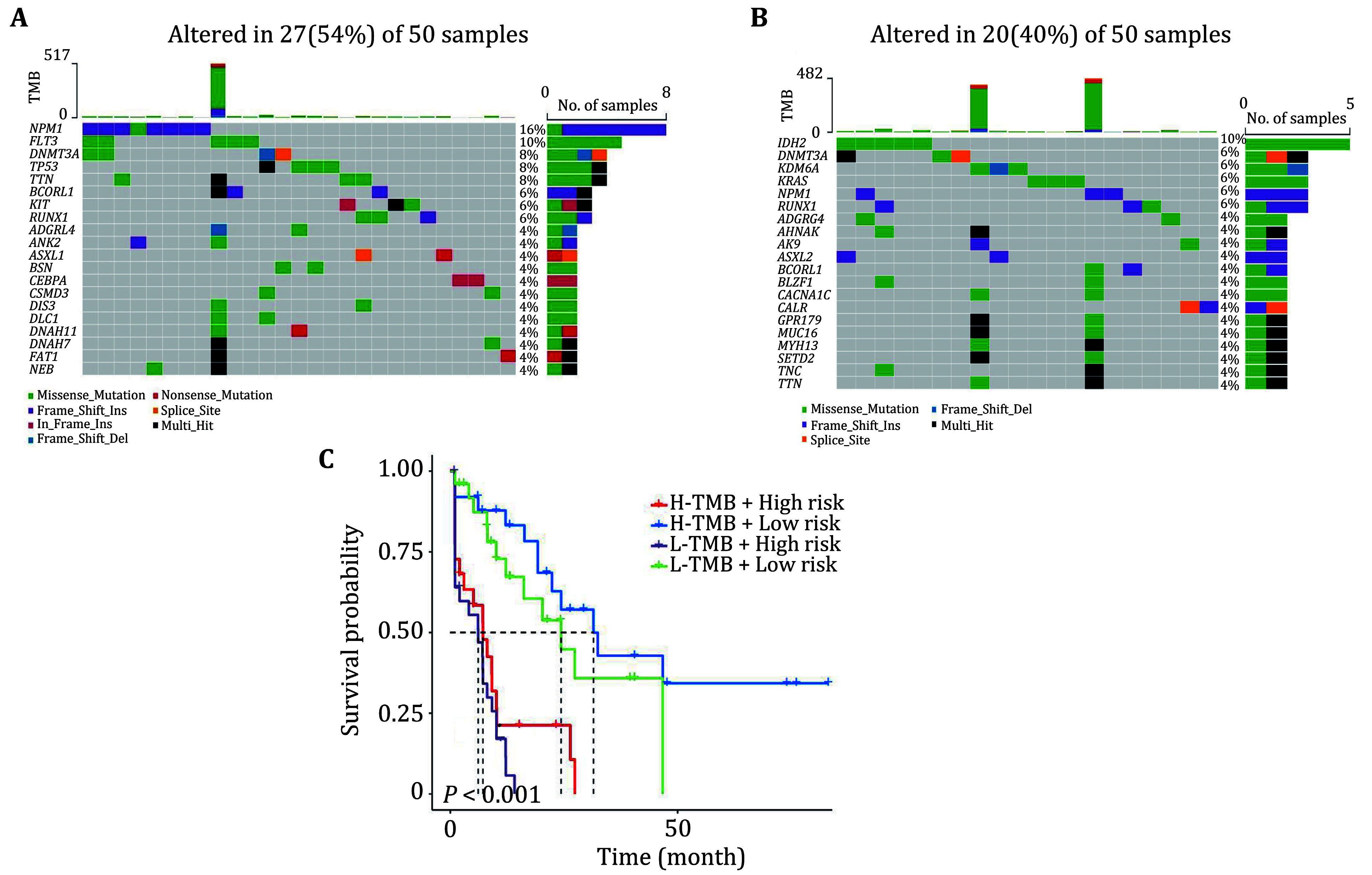
Analysis of somatic mutation landscape. **A** The top 20 mutated genes in the high risk group. **B** The top 20 mutated genes in the low risk group. **C** The survival curves of high and low TMB groups combined with high and low risk groups

AML is a malignant hematological tumor that is highly heterogeneous. It often presents multiple gene mutations that significantly alter the tumor's biological characteristics, and affects the treatment and prognosis of patients. Therefore, we analyzed somatic mutations in the high and low risk groups. It was found that the frequency of mutations in the high risk group (54%) was higher than that in the low risk group (40%). Genes common to both groups were also found to be mutated more frequently in the high risk group, for example, TTN (8% vs. 4%) and DNMT3A (8% vs. 6%). Some mutated genes that were only present in the high risk group might be associated with poorer prognosis, such as TP53 and FLT3 (Fig. 5A and 5B). In addition, the predictive ability of the risk score model for Tumor Mutation Burden (TMB) was assessed by categorizing patients into four groups based on the median risk score versus median TMB. The KM survival curves indicated a more significant survival advantage and better prognosis for patients in the high TMB+ low risk group (Fig. 5C).

### Enrichment analysis of high- and low-risk groups

**Figure 6:**
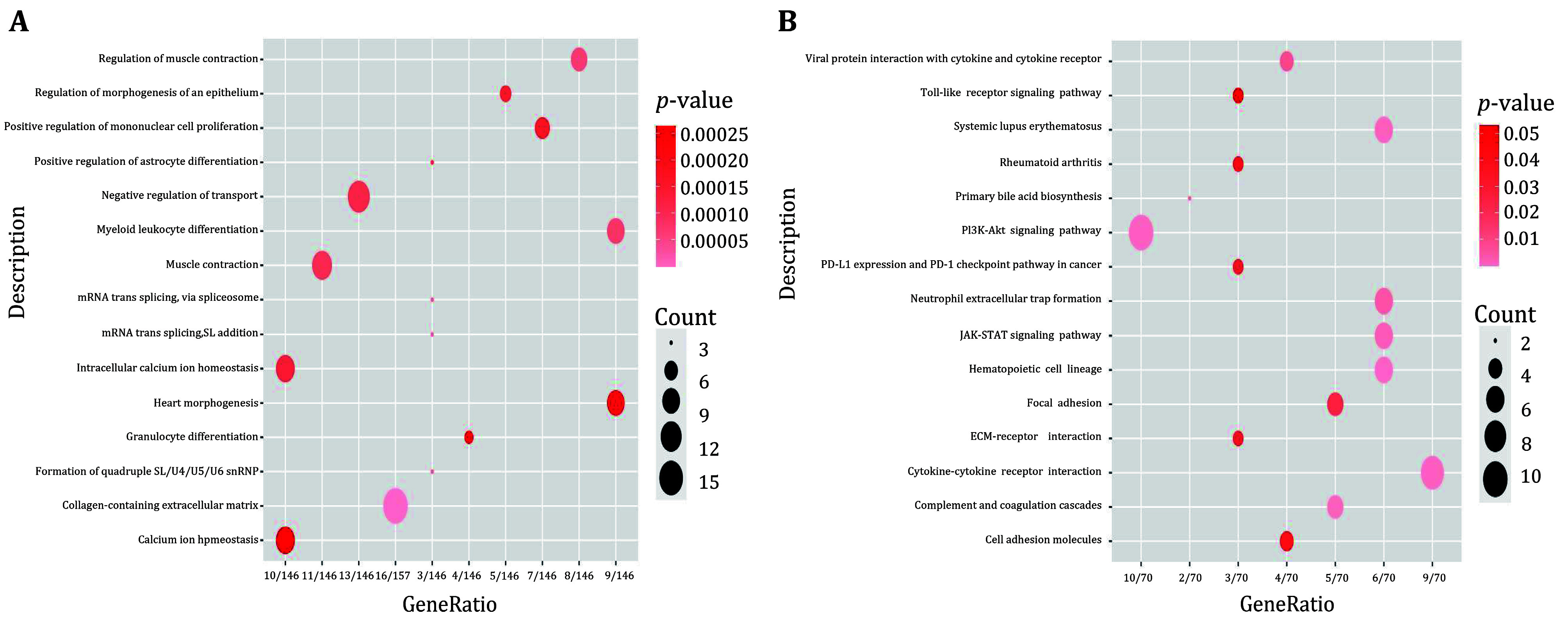
Functional enrichment analysis of differentially expressed genes in the high and low risk group. **A** GO enrichment analysis. **B** KEGG enrichment analysis. The depth of the color of the circle represents the size of the *p*-value, and the size of the circle represents the amount of gene enrichment in panels A and B

To investigate the role of risk models in AML, we performed GO and KEGG enrichment analyses using the differentially expressed genes in the high and low risk groups. The results of GO enrichment analyses showed that the differentially expressed genes were enriched in myeloid leukocyte differentiation, calcium homeostasis, granulocyte differentiation, and other biological functions (Fig. 6A). The KEGG enrichment analyses revealed that the differentially expressed genes were enriched in several pathways, including cytokine-cytokine receptor interactions, toll-like receptor signaling pathway, transcriptional dysregulation in cancer, hematopoietic cell lines, viral proteins interacting with cytokines and cytokine receptors, and ECM-receptor interactions (Fig. 6B). The enrichment analysis results indicate that the differentially expressed genes were enriched in immune-related and cancer-related pathways. This suggests that FerRLSig may play a crucial role in cancer development and immune microenvironment regulation, providing a rationale for the immune analysis in this risk model.

### Analysis of the tumor immune microenvironment

**Figure 7:**
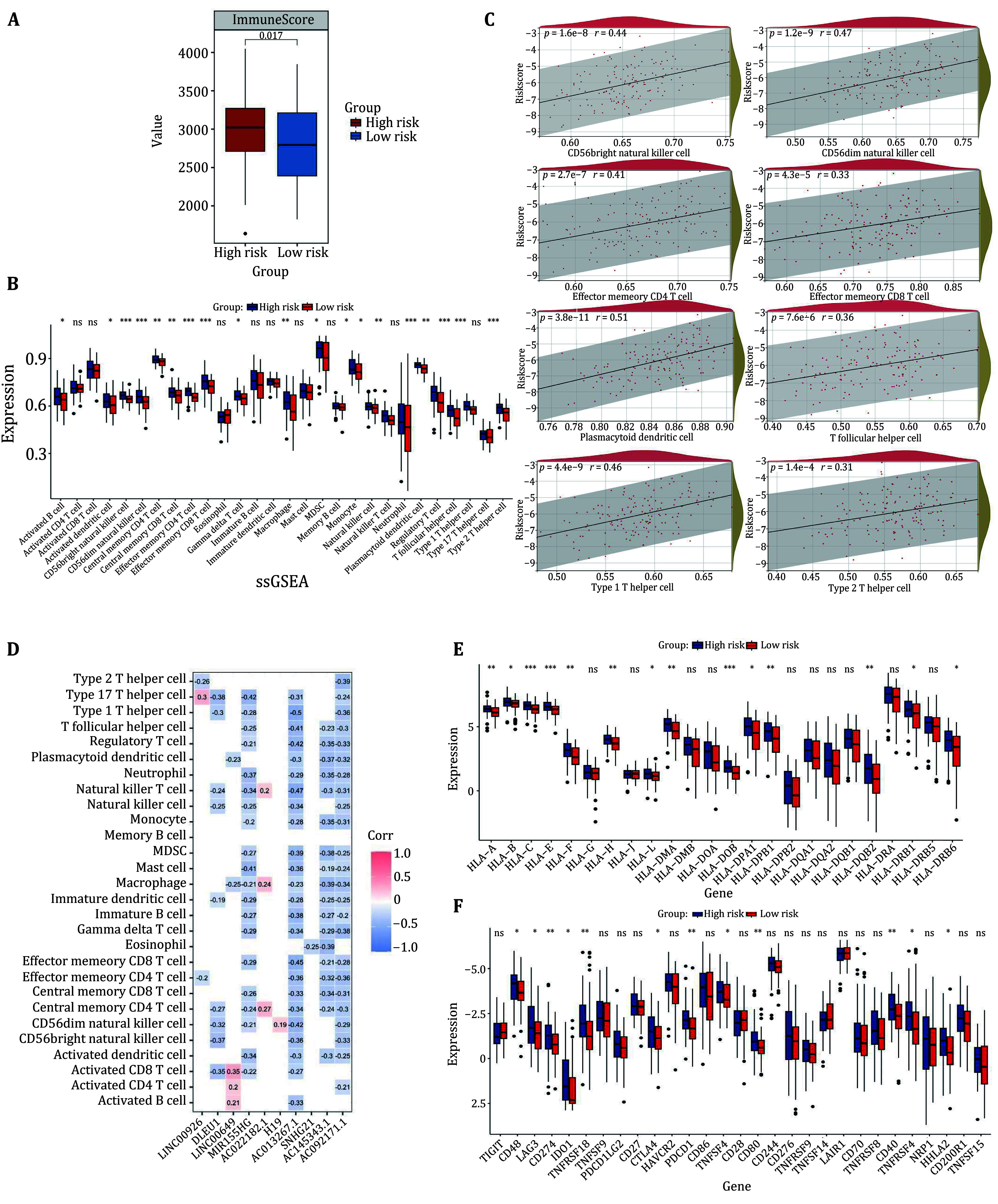
The tumor-infiltrating immune cell correlation with the risk model. **A** Immune score in the high and low risk groups. **B** Infiltration levels of 28 immune cells in the high and low risk groups. **C** Analysis of correlation between risk score and immune cells. **D** Correlations between the abundance of 28 immune cells and ten genes in a prognostic model. **E** Gene expression analysis of HLA genes. **F** Gene expression analysis of immune checkpoint genes. *p < 0.05; **p < 0.01; ***p < 0.005; ns, not significant

The tumor microenvironment plays a crucial role in a patient's disease progression and significantly affects the effectiveness of immunotherapy and overall patient survival. We study the tumor immune microenvironment in high risk and low risk groups. It was found that patients in the high risk group had higher immune scores than those in the low risk group, and the low risk group had less immune infiltration than the high risk group (Fig. 7A). In addition, we compared 28 immune cells between the high and low risk groups using the ssGSEA algorithm. The study results indicate that immune cells with a *p*-value < 0.05 had a higher infiltration rate in the high risk group compared to the low risk group. Among them, eight immune cells were found to be the most significant: CD56 bright natural killer cells, CD56 dim natural killer cells, effector-memory CD4 T cells, effector-memory CD8 T cells, plasma cell-like dendritic cells, T follicular helper cells, type 1 T helper cells and type 2 T helper cells (Fig. 7B). The association between the risk score and eight immune cells were explored. The significant positive correlations between the risk score and these immune cells were found (Fig. 7C). This finding provides important clues for further understanding the pathogenesis and prognosis of AML. We also evaluated the correlation between the ten prognostic genes and the abundance of immune cells. The results indicate that over 50% of the immune cells were linked to the ten prognostic genes (Fig. 7D).

We compared the expression of human leukocyte antigen (HLA) genes related in the high risk and low risk groups and found that most HLA-related genes were up-regulated in the high risk group (Fig. 7E). In addition, the relationships between 29 immune checkpoints and risk groups were also investigated, and it was found that the expressions of 12 immune checkpoint genes were lower in the low risk group than in the high risk group (Fig. 7F). The results indicate that the degree of immune infiltration is positively correlated with the expression of immune checkpoint genes. These results suggest that the high risk group is more prone to immune escape, and it is possible that patients treated with ICI (immune checkpoint inhibitors) have a better prognosis.

## DISCUSSION

In this study, the ten ferroptosis-related lncRNA LINC00926, DLEU1, LINC00649, MIR155HG, AC0022182.1, H19, AC013267.1, SNHG21, AC145343.1 and AC092171.2 were obtained in the prognostic model. It was found that LINC00926, DLEU1, LINC00649, AC013267.1 and AC145343.1 are protective factors, and MIR155HG, AC0022182.1, H19, and SNHG21 are risk factors. Some lncRNA has been found to play a role in cancer, *e*.*g*., in breast cancer, FOXO3A-induced LINC00926 can inhibit tumor cell growth and metastasis by suppressing the PGK1-mediated Warburg effect (Chu *et al.* 2021). The gene DLEU1 regulates the NF-kB signaling pathway, inducing or inhibiting its activity, and the activation of NF-kB had previously been shown to promote leukemia cell survival, suggesting that DLEU1 can influence leukemogenesis (Garding *et al.* 2013). Under-expression of gene LINC00649 is a poor prognostic marker for AML (Guo *et al.* 2020). MYB physically binds to the promoter of the miR-155 host gene (MIR155HG) and stimulates its transcription, which leads to leukemia (Vargova *et al.* 2011). H19 overexpression promotes leukemia and predicts poor prognosis in AML (Zhang *et al.* 2018). H19 is activated by binding to hsa-mi-19a and hsa-miR-19b to regulate ID2 expression, which may play a role in AML cell proliferation (Zhao *et al.* 2017). H19 inhibits apoptosis in AML cells by targeting miR-29a-3p (Zhao and Liu 2019). The gene SNHG21 is a low risk gene in hepatocellular carcinoma (Yang *et al.* 2022). The replacement version AC145343.2 was identified as a prognostic factor for glioma mesenchymal transition (Liang *et al.* 2020). Our study suggests that the ten ferroptosis-related lncRNA plays an important role in the development and progression of AML, which will be helpful for future research on AML.

In addition, we identified some mutated genes that were associated with poor prognosis of patients in the high-risk group of AML, such as TP53 and FLT3. Fms-like tyrosine kinase 3 (FLT3) is a member of the receptor tyrosine kinase III family (Zhong *et al.* 2020). FLT3 mutations are an important molecular marker in a variety of cancers such as AML. FLT3 mutations mainly include internal tandem repeat mutations (ITD) and point mutations in the activation loop of the kinase region (TKD) (Daver *et al.* 2019). FLT3-ITD is a common driver mutation that manifests as a high leukemic burden and leads to poor prognosis in patients with acute myeloid leukemia (Staudt *et al.* 2018). In FLT3-TKD, the prognostic value of FLT3 mutations, which are less common in AML, is uncertain (Daver *et al.* 2019). NPM1 is a member of the nucleophosmin family of proteins, which play an important shuttling role between the nucleus and the cytoplasm and are involved in a wide range of cellular functions as chaperone proteins (Karimi Dermani *et al.* 2021). This protein is widely expressed in the nucleolus and plays a critical role in maintaining normal physiological cellular function. NPM1 mutant AML is a clinically heterogeneous group as it is almost always present in the context of other mutations (Ley *et al.* 2013). NPM1 mutations usually occur in conjunction with FLT3, DNMT3A, or other mutations that promote leukemogenesis (Papaemmanuil *et al.* 2016).

## MATERIALS AND METHODS

### Database construction

We downloaded RNA-seq data, clinical patient information, and somatic mutation data for AML in the TCGA database (https://www.cancer.gov/). Because the 151 samples in the RNA-sequencing data did not include normal samples, we obtained RNA-seq transcriptome raw data for 70 myeloid tissue samples through the GTEx database (https://gtexportal.org/home). Finally, the samples were divided into training and testing sets in a ratio of 6:4. Ferroptosis-related genes were obtained from the FerrDb database, MSigDB database and the ncFO database, and a total of 572 ferroptosis-related genes were obtained after de-redundancy.

### Identification of prognostic ferroptosis-related lncRNA

The differentially expressed ferroptosis-related genes and lncRNA between tumor samples and normal samples were screened separately by a limma R package, and the differential ferroptosis-related genes and lncRNA were screened according to the conditions of |log_2_(fold change)| > 1, *p* < 0.05. Pearson correlation analysis was used to evaluate the ferroptosis-related lncRNA, and the lncRNA that satisfied the conditions |R| > 0.3, *p* < 0.001 were selected as ferroptosis-related lncRNA. Then, the prognosis-related ferroptosis-related lncRNA was screened again by the results of the univariate Cox analysis, least absolute shrinkage and selection operator (LASSO) analysis, and multivariate Cox regression analysis. The risk scores for each sample were calculated based on the results of the multivariate Cox regression analysis and the expression of ferroptosis-related lncRNA.

### Validation of prognostic models

Patients were categorized into the high and low risk groups using the median risk value as a criterion. We used Kaplan-Meier (KM) analysis and the ROC analysis to assess the predictive effect of prognostic models for AML prognosis (Wang *et al.* 2023). The survival rates for different risk groups were calculated by using the model's risk score based on KM analysis. The area under the ROC curve shows the accuracy of the model over a specific time period (such as three, and five years). The risk scores in AML patients were determined by using univariate logistic regression analysis, multivariate logistic regression analysis and PCA, and they can serve as independent predictors.

### Somatic mutation analysis

Gene mutation profiles and tumor mutational load (TMB) in high and low risk groups were analyzed by using somatic mutation data from the TCGA database for AML. Patients were grouped according to the median TMB value.

### Immune infiltration analysis

The goal of immune infiltration analysis is to gain an in-depth understanding of the composition of immune cells in the body's microenvironment so as to accurately identify the types of immune cells that play a key role in the development and progression of disease. In this paper, high and low risk groups were analyzed by using the ssGSEA algorithm, which uses gene expression values to assess the relative abundance of 28 immune cell types in the infiltrate.

## Conflict of interest

Shuhan Liu, Yingli Chen, Qianzhong Li, Zhiyu Fan, Menglan Li and Pengyu Du declare that they have no conflict of interest.
